# Diagnosis, Treatment, and Outcomes in Children With Congenital Nephrogenic Diabetes Insipidus: A Pediatric Nephrology Research Consortium Study

**DOI:** 10.3389/fped.2019.00550

**Published:** 2020-01-21

**Authors:** Cynthia D'Alessandri-Silva, Melinda Carpenter, Rose Ayoob, John Barcia, Aftab Chishti, Alex Constantinescu, Katherine M. Dell, Julie Goodwin, Shireen Hashmat, Sandra Iragorri, Cristin Kaspar, Sherene Mason, Jason M. Misurac, Melissa Muff-Luett, Christine Sethna, Shweta Shah, Patricia Weng, Larry A. Greenbaum, John D. Mahan

**Affiliations:** ^1^Division of Nephrology, Connecticut Children's Medical Center, Hartford, CT, United States; ^2^Department of Pediatrics, University of Connecticut Health Center, Farmington, CT, United States; ^3^Department of Research, Connecticut Children's Medical Center, Hartford, CT, United States; ^4^Department of Nephrology, West Virginia University-Charleston, Charleston, WV, United States; ^5^Department of Pediatrics, University of Virginia, Charlottesville, VA, United States; ^6^Division of Nephrology, Hypertension and Renal Transplantation, University of Kentucky, Lexington, KY, United States; ^7^Joe DiMaggio Children's Hospital, Hollywood, FL, United States; ^8^Center for Pediatric Nephrology, Cleveland Clinic Children's and Case Western Reserve University, Cleveland, OH, United States; ^9^Department of Pediatrics, Yale University School of Medicine, New Haven, CT, United States; ^10^Department of Pediatrics, University of Chicago, Chicago, IL, United States; ^11^Division of Nephrology and Hypertension, Department of Pediatrics, Oregon Health & Science University, Portland, OR, United States; ^12^Pediatric Nephrology, Virginia Commonwealth University, Children's Hospital of Richmond, Richmond, VA, United States; ^13^Division of Pediatric Nephrology, Dialysis and Transplantation, University of Iowa Stead Family Department of Pediatrics, Iowa City, IA, United States; ^14^Division of Pediatric Nephrology, University of Nebraska Medical Center, Omaha, NE, United States; ^15^Division of Pediatric Nephrology, Department of Pediatrics, Cohen Children's Medical Center, New Hyde Park, NY, United States; ^16^Renal Section, Department of Pediatrics, Baylor College of Medicine, Houston, TX, United States; ^17^Department of Pediatric Nephrology, UCLA Medical Center and UCLA Medical Center-Santa Monica, Los Angeles, CA, United States; ^18^Division of Pediatric Nephrology, Emory University and Children's Healthcare of Atlanta, Atlanta, GA, United States; ^19^Department of Nephrology, Nationwide Children's Hospital, The Ohio State University, Columbus, OH, United States

**Keywords:** nephrogenic diabetes insipidus (NDI), pediatric, multicenter, vasopressin (AVP), genetic disease

## Abstract

**Background and Objectives:** Congenital or primary nephrogenic diabetes insipidus (NDI) is a rare genetic disorder that severely impairs renal concentrating ability, resulting in massive polyuria. There is limited information about prognosis or evidence guiding the management of these patients, either in the high-risk period after diagnosis, or long-term. We describe the clinical presentation, genetic etiology, treatment and renal outcomes in a large group of children <21 years with NDI.

**Design:** A multi-center retrospective chart review.

**Results:** We report on 66 subjects from 16 centers. They were mainly male (89%) and white (67%). Median age at diagnosis was 4.2 months interquartile range (IQR 1.1, 9.8). A desmopressin acetate loading test was administered to 46% of children at a median age of 4.8 months (IQR 2.8, 7.6); only 15% had a water restriction test. Genetic testing or a known family history was present in 70% of the patients; out of those genetically tested, 89 and 11% had mutations in *AVPR2* and *AQP2*, respectively. No positive family history or genetic testing was available for 30%. The most common treatments were thiazide diuretics (74%), potassium-sparing diuretics (67%) and non-steroidal anti-inflammatory drugs (42%). At the time of first treatment, 70 and 71% of children were below −2 standard deviations (SD) for weight and height, respectively. At last follow-up, median age was 72.3 months (IQR 40.9, 137.2) and the percentage below −2 SD improved to 29% and 38% for weight and height, respectively. Adverse outcomes included inpatient hospitalizations (61%), urologic complications (37%), and chronic kidney disease (CKD) stage 2 or higher in 23%.

**Conclusion:** We found the majority of patients were treated with thiazides with either a potassium sparing diuretic and/or NSAIDs. Hospitalizations, urologic complications, short stature, and CKD were common. Prospective trials to evaluate different treatment strategies are needed to attempt to improve outcomes.

## Introduction

Congenital nephrogenic diabetes insipidus (NDI) is a rare disorder with an unknown prevalence, although a report from Quebec estimated a prevalence in males of 8.8: 1,000,000 (1). NDI prevents the kidneys from concentrating urine by impairing the collecting duct's ability to respond to vasopressin. Inheritance is x-linked in 90% of patients due to mutations in the gene coding for the vasopressin type 2 receptor (*AVPR2*) (1). The remaining 10% of patients have autosomal recessive forms due to mutations in the gene coding for the water channel aquaporin 2 (*AQP2*) (2).

Massive free water losses cause significant morbidity, even in treated patients (3). High fluid intake is necessary to avoid hypernatremic dehydration, which can result in permanent neurologic complications. Patients are also at risk for nocturnal enuresis, hydronephrosis, and poor growth, presumably due to the need for high water consumption that interferes with adequate caloric intake. A variety of medications, including thiazide diuretics, potassium sparing diuretics (PSD), and non-steroidal anti-inflammatory drugs (NSAIDs)/Cox-2 inhibitors have been utilized to decrease urine output, although their efficacy is suboptimal (3).

There have been few long-term studies on patients with NDI that provide information on clinical outcomes, disease complications or typical therapeutic approaches. Parents of these children frequently request information regarding prognosis and future renal outcomes. The rarity of congenital NDI has limited the size and ability to perform studies to address long-term outcomes. Hence, we utilized a multicenter strategy to address this gap in knowledge and assemble a large cohort for investigation.

## Materials and Methods

### Research and Design

A multicenter, retrospective chart review was conducted after local Institutional Review Board approval at participating institutions from the Pediatric Nephrology Research Consortium (PNRC). Data were collected via medical record review. Study data were collected and managed using REDCap (Research Electronic Data Capture) tools hosted at the University of Connecticut (4). REDCap is a secure, web-based application designed to support data capture for research studies. Measures of central tendency were used to describe characteristics, management, and outcomes of the population. Median and interquartile ranges were reported for skewed data.

### Subjects and Outcome Definitions

Patients ≤21 years of age and diagnosed with NDI via genetic testing and/or water restriction/DDAVP loading tests between January 1, 1995 and December 31, 2015 were included. Data collected included: (1) demographic (date of birth, gender, ethnicity/race), (2) diagnostic evaluation (age at diagnosis, diagnostic tests, and laboratory findings), (3) genotype (gene and type of mutation), (4) nutritional history (formula, dietician involvement, gastrostomy tube use), (5) medications (type, start and stop dates, and reason for starting or stopping), (6) laboratory findings at diagnosis and at last follow-up (serum osmolality, sodium, creatinine), and (7) outcomes (height, weight, hospitalizations and indications, disease complications, treatment complications). Medications were grouped into three categories: (1) thiazide diuretics (hydrochlorothiazide, chlorthalidone, and chlorothiazide), (2) potassium-sparing diuretics (amiloride and spironolactone), and (3) non-steroidal anti-inflammatory drugs (indomethacin, naproxen, and ibuprofen). World Health Organization (WHO) growth charts were used to determine height and weight standard deviation values for children up to age 2 years (5). CDC growth charts were utilized for children 2 years and older (6). Current chronic kidney disease (CKD) stage was based on KDIGO staging guidelines, with the estimated glomerular filtration rate (eGFR) determined by the modified Schwartz Equation (7, 8).

### Statistical Methods

The Statistical Package for Social Sciences (SPSS) version 17 was used for data analysis. Median and interquartile ranges were used for skewed data.

## Results

### Patient Characteristics and Genetic Variants

There were 66 subjects (89% male) from 16 participating PNRC centers. Patient characteristics are shown in Table 1. Out of those with a known mutation, 89% revealed an *AVPR2* mutation and 11% revealed *AQP2*. There were two sets of affected twins; one set had *AVPR2* mutations and one set had *AQP2* mutations.

**Table 1:** Demographic, diagnostic, and nutritional support data.

		**N (%) (*n* = 66)**	**Median months (IQR)**
**Demographics**			
Race	White	44 (67)	
	Black	9 (14)	
	Other or unknown	7 (10)	
	Asian	6 (9)	
Hispanic ethnicity		12 (18)	
Gender	Male	59 (89)	
	Female	7 (11)	
**Diagnostic features**			
Age at diagnosis			4.2 (1.2, 9.8)
High serum osmolality		44 (67)	
Low urine osmolality		46 (70)	
ADH level		30 (46)	1.1 (0.5, 1.6)
MRI of head		24 (36)	
**Genetic information**			
Positive family history with unknown mutation		6 (9)	
Known mutations:			
*AVPR2*	34 (52)		
*AQP2*	4 (6)		
Unclear significance	2 (3)		
Unknown (no family history or genetic testing)		20 (30)	
**Nutritional supports**			
Ever breast fed		34 (52)	
Formula fed		53 (80)	
Dietician involvement		43 (65)	5.2 (2.0, 11.1)
Low sodium diet recommended		35 (53)	
Formula prescribed		41 (62)	
	SIM PM 60/40	20 (30)	
	Other	21 (32)	
Nutrient supplementation		22 (33)	
G-tube placement		24 (36)	
	Start age		6.6 (3.5, 14.2)
	Stop age		51.1 (34.5, 68.4)
Number of months with			39.0 (21.1, 56.5)

### Diagnostic Evaluations

The median age at diagnosis was 4.2 months (IQR 1.2, 9.8) and 67% of patients had elevated serum osmolality at diagnosis (>300 mOsm/L). Fifteen percent had a water restriction test at a median age of 16.2 months (IQR 5.6, 35.6) and 46% had a desmopressin acetate loading test at a median age of 4.8 months (IQR 2.8, 7.5). A subset of 30 patients had ADH measured at a median age of 1.2 months (IQR 0.5, 1.6). Twenty four patients (36%) underwent cranial MRI as part of their diagnostic evaluation. Out of these 24 patients, two had absence of posterior pituitary bright spot, one had matter volume loss of the lateral ventricles and thin corpus callosum, two had evidence of previous ischemic insult and one was an incomplete study due to motion artifact.

### Nutritional Supports and Growth

Most patients were supplemented with formula during infancy or childhood (80%). Over half (52%) received some breast milk. Evidence of exclusive breast feeding was not queried. For the entire cohort, 62% received a prescribed formula with a decreased solute load such as Similac PM 60/40 (30%). A dietician was involved in 65% of patients, with first contact occurring at median age 5.2 months (IQR 2.0, 11.0). A low salt diet was recommended to 53% of patients and nutritional supplementation was prescribed to 33%, with four patients receiving Duocal and three drinking Pediasure. Others reported receiving MCT oil (*n* = 1), Safflower oil/Beneprotein (*n* = 2), and Polycose (*n* = 1).

A gastrostomy tube (g-tube) was placed in 36% of patients at median age 6.7 months (IQR 3.5, 14.2). Failure to thrive (FTT) was the most common reason for g-tube placement, although poor oral intake was also noted. G-tubes were left in place for a median 39.0 months (IQR 21.0, 56.5).

**Figure 1:**
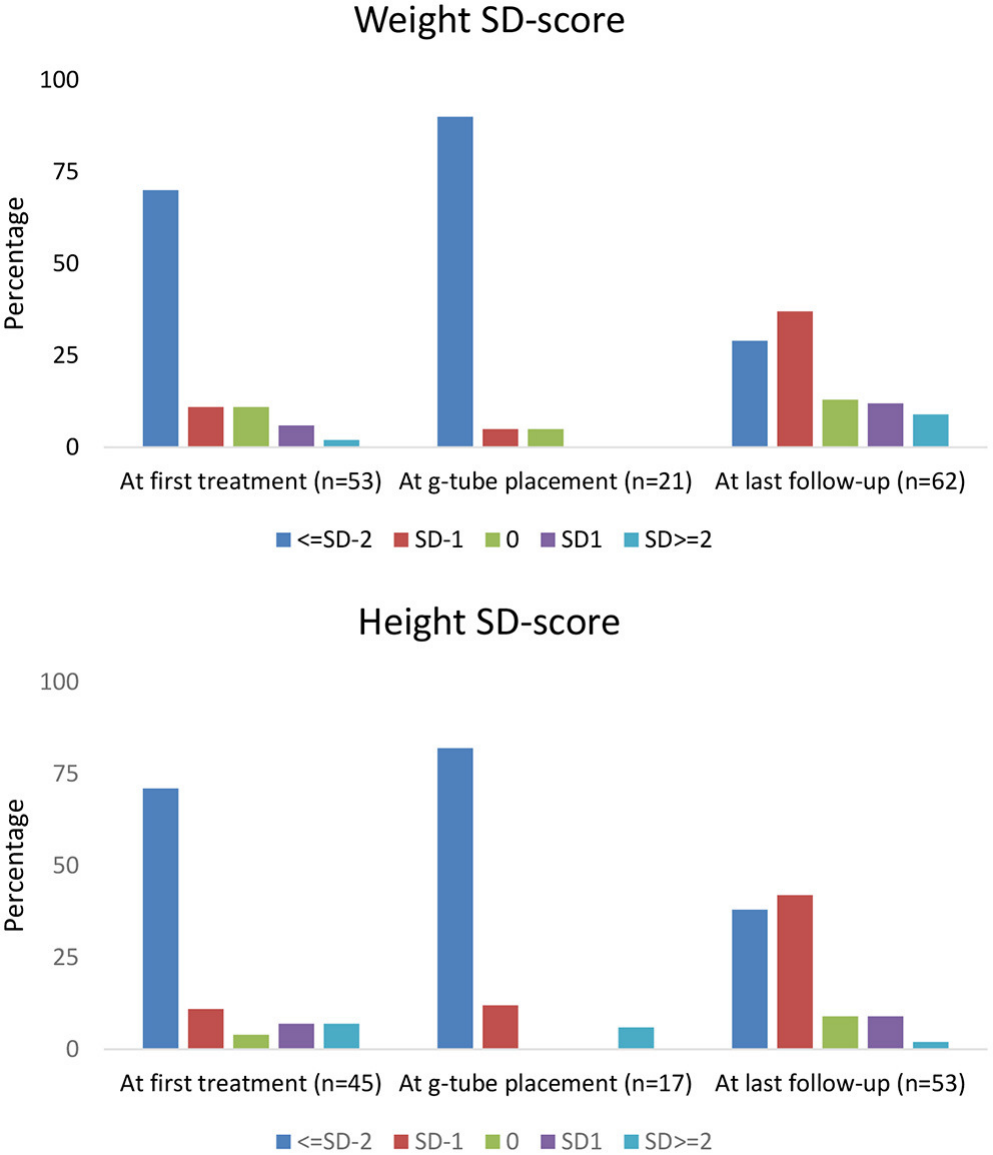
Percentage of children within each height and weight standard deviation category.

Height and weight standard deviation (SD) scores are reported in Figure 1. Weight below −2 SD was present in 70% of children at time of first treatment, and in 90% at g-tube placement. Weight below −2 SD was present in only 29% at last follow-up. Height below −2 SD was present in 71 and 82% of children at time of first treatment and g-tube placement, respectively. Height below −2 SD was present in only 38% at time of last follow-up.

### Treatment Regimens

**Figure 2:**
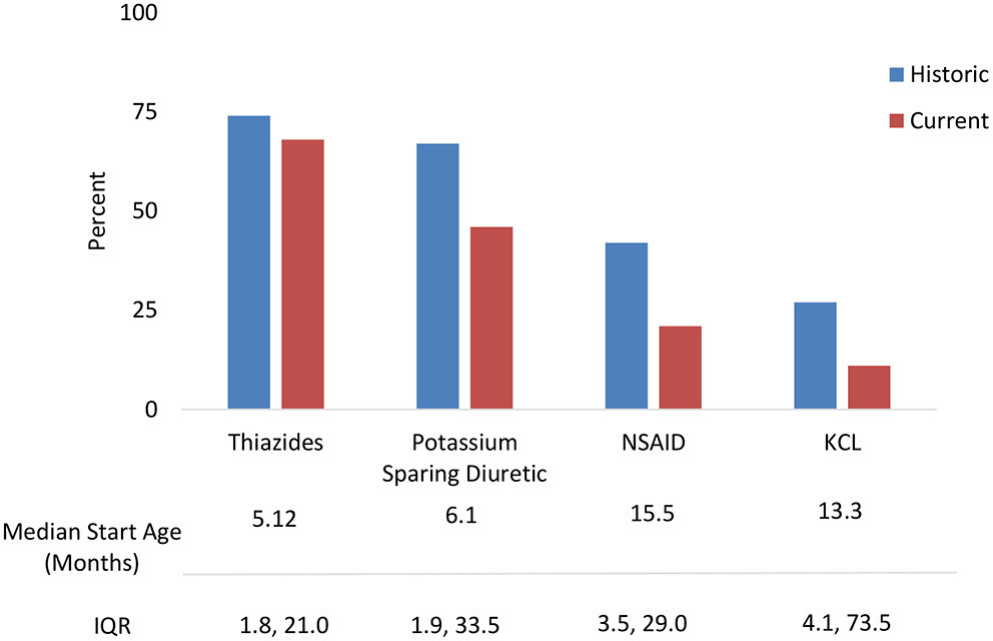
NDI treatments: Historic use and current use of standard medications with median start age.

Treatments were analyzed for current use and historic use (Figure 2). Thiazides were prescribed to 74% of patients, followed by PSD (67%) and NSAIDs (42%). Thiazides were the first treatment in the majority of children, with median start age 5.1 months (IQR 1.8, 21.0). PSD and NSAIDs had median start age 6.0 months (IQR 1.8, 33.4) and 15.5 months (IQR 3.5, 29.0), respectively. Potassium supplementation was used in 27%, with median start age 13.3 months (IQR 4.1, 73.5). A small percentage used growth hormone (5%) and angiotensin-converting enzyme (ACE) inhibitors (3%).

The most frequent combination treatments were thiazide with PSD (33%), a thiazide with PSD and NSAID (17%), a thiazide and NSAID (15%), or PSD with NSAID (3%). NSAIDS were discontinued for the following reasons including increased serum creatinine (18%), gastrointestinal bleeding (11%), and general concerns about long-term use (7%).

### Serum Chemistries and Renal Function Outcomes

Table 2 provides the eGFR and serum sodium at various time points. Median serum sodium at first treatment was 151 mmol/L (IQR 141, 158), with eGFR of 25 ml/min/1.73 m^2^ (IQR 23, 33). At last follow-up, median age was 72.3 months (IQR, 40.9, 137.2) and median eGFR was 103 ml/min/1.73 m2 (IQR 86, 116). Serum sodium was not available at last follow-up.

**Table 2:** Serum chemistries and renal function outcomes.

	**At first treatment/intervention**		**At last follow-up**		**At first inpatient admission**	
	***n***	**Median (IQR)**	***n***	**Median (IQR)**	***n***	**Median (IQR)**
Sodium	49	151 (141, 158)		N/A	35	151 (144, 158)
Age in months	59	4.6 (1.8, 11.4)	65	72.3 (40.9, 137.2)	38	4.9 (1.2, 49.2)
eGFR	33	25 (23, 33)	61	103 (86,116)	27	68 (38, 98)

Based on eGFR, CKD stage 2 or higher was noted in 30% of patients at time of last follow-up-up as indicated in Table 3. Of the CKD stage 2 patients, 56% (*n* = 9) had the *AVPR2* mutation and 6% (*n* = 1) had the *AQP2* mutation. Abnormal renal ultrasounds were identified in four of these patients. Findings included bilateral mild hydronephrosis (*n* = 1), increased cortical echogenicity (*n* = 1), large kidneys with hyperechoic pyramids (*n* = 1), and moderately distended bladder (*n* = 1). The one patient with CKD stage 3 was 20 years old at time of last follow-up, had the *AVPR2* mutation and renal ultrasound findings included both kidneys small in size (4th and 6th % for length) with prominence of ureters noted. One patient started dialysis at just over 1 year of age, genetic mutation unknown with renal ultrasound demonstrating increased echogenicity of both kidneys, found to be small in size.

**Table 3:** Inpatient admissions, renal outcomes, and follow-up data.

		***N* (%)**	**Median number of months (IQR)**
Outcomes	**Inpatient admission**	40 (61)	
	Number of inpatient admissions		
	0	26 (39)	
	1	22 (33)	
	2	7 (11)	
	3	2 (3)	
	4	9 (14)	
	First inpatient admission age		4.9 (1.2, 6.4)
	Length of stay		0.33 (0.13, 0.5)
	CKD/eGFR at time of last visit^*^		
	Stage 1: ≥90 mL/min/1.73 m^2^	43 (70)	
	Stage 2: 60–89 mL/min/1.73 m^2^	16 (26)	
	Stage 3: 30–59 mL/min/1.73 m^2^	1 (2)	
	Stage 4: 15–29 mL/min/1.73 m^2^	0 (0)	
	Stage 5: <15 mL/min/1.73 m^2^	1 (2)	
Follow-up	**Age in months at time of last visit**		72.3 (40.9, 137.2)
	**Renal ultrasound findings**		
	Hydronephrosis	12 (18)	
	Bladder distension/trabeculation	5 (8)	
	Renal dysplasia	9 (14)	
	**Enuresis at last visit**	29 (44)	
	**Dialysis**	1 (2)	
	Age in months at first dialysis	13	

* *Values available for 61 subjects*.

**Figure 3:**
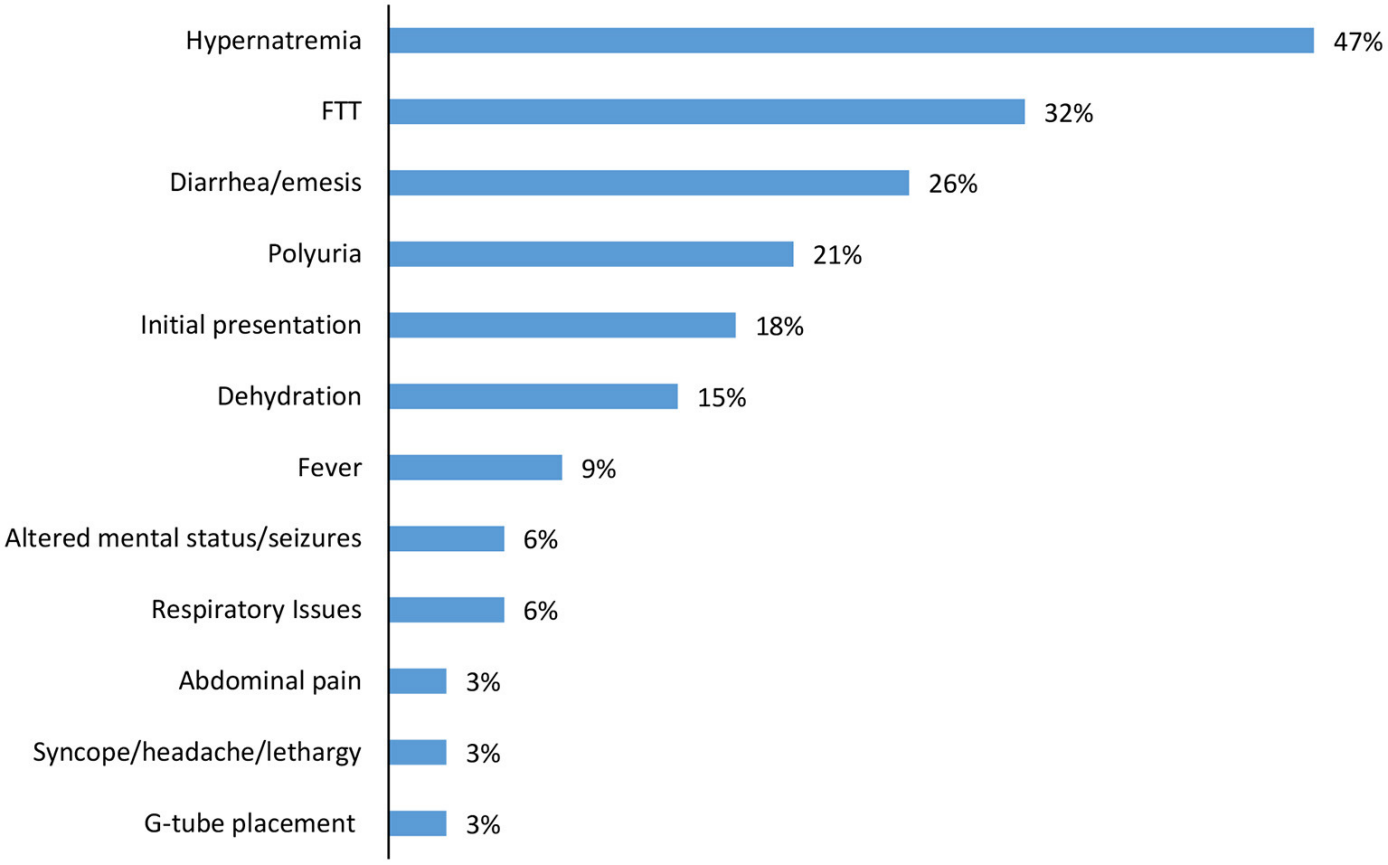
Diagnoses at first inpatient admission*. *Children could have >1 diagnoses.

Over half of the children had one or more inpatient admission (61%), with median admission age 4.9 months (IQR 1.2, 6.4) and length of stay of 10 days (IQR 4, 15). Hypernatremia (47%) and failure to thrive (32%) were the most common indications for admission, with diarrhea/emesis (26%) and polyuria (21%) also noted (Figure 3). In addition to initial hospitalization, 28% of the cohort had a subsequent hospital admission.

### Urologic Findings

Urologic findings occurred in 37% of children (*n* = 23, Table 3). Abnormal renal ultrasound findings included unilateral/bilateral hydronephrosis (18%, 10/12 *AVPR2*), renal dysplasia (14%, 3/5 *AVPR2*), and bladder distension and/or trabeculation (8%, 4/9 *AVPR2*). Increased bladder capacity was noted in four children. At last clinic visit, which occurred at median age 6 years (IQR 3, 11), enuresis was reported in 44% of children.

## Discussion

This study describes complications and treatment approaches in a large contemporary cohort of children with NDI. Long-term morbidities caused by NDI in this cohort include primary nocturnal enuresis (44%), persistent small stature (38%), urologic complications (37%), persistent failure to thrive (29%), and CKD stage 2 or greater (30%). Treatment included unrestricted intake of free water, aggressive enteral nutrition, thiazide diuretics, PSD, and NSAIDs. Thiazides and PSDs were the most common combination for medical therapy.

There has been limited reporting of CKD in children with NDI, though CKD has been reported in other tubular disorders due to complications such as nephrocalcinosis and non-steroidal anti-inflammatory drug toxicity (9, 10). In 2018, Sharma et al. reported a single center experience with NDI in 33 children. Their median length of follow-up was 9.5 years and these patients were similar to ours in that they presented at median age 0.6 years. At last follow-up, 97% were classified as CKD stage 1 or 2 based on eGFR (11). In our cohort, 30% of patients had CKD stage 2 or greater, with one unusual case of a patient on dialysis (CKD stage 5). Data on number of hospitalizations and length of treatment with NSAIDs that might have helped shed light on the different outcomes between that study and ours are not available but these differences highlight the need for more detailed, even prospective, documentation of these subjects' courses.

Episodes of hypernatremic dehydration are well-described in the course of NDI and place patients at risk of acute kidney injury (AKI). Published data on rates of AKI in this population are limited. A limitation of our study is the lack of serial creatinine measurements available during hospitalizations, thus preventing us from accurately reporting and assessing the frequency and severity of AKI episodes. It is possible that episodes of AKI contributed to the development of CKD in our cohort, but future prospective studies will be needed to examine this hypothesis.

Over half of the patients in our cohort experienced hospitalizations directly related to NDI. Diagnosis at the time of admission included hypernatremia, diarrhea and /or vomiting causing dehydration and polyuria, all of which place children at risk of AKI (12). The fact that that almost a third of our cohort experienced multiple hospitalizations underscores the increased morbidity caused by NDI.

Urologic abnormalities have previously been described in children with NDI and are thought to be secondary to longstanding polyuria. Megacystis, trabeculated bladder, hydronephrosis, and large hypotonic bladder have been identified (13, 14). In our cohort, 44% of our patients had enuresis at last follow-up, with 21% being aged 8 or older. Prolonged nocturnal enuresis, often past age 10 years, has previously been described in many of these children (15). Additionally, 26% of our patients had hydronephrosis and/or an abnormal bladder by renal ultrasound. Bladder dysfunction related to polyuria has been described in other studies of affected children (16–18).

With respect to growth, previous studies in NDI patients revealed short stature at diagnosis, with catch-up growth occurring by school age (9). In our cohort, the majority of children measured −2 SD or more below height and weight at initial treatment. However, at the last follow-up visit, 29% of children remained below −2 SD for weight and 38% were below −2 SD for height. These observations demonstrate that current treatment with nutritional support and normalization of electrolyte abnormalities may assist in catch-up growth in many children with NDI; yet, in a sizeable proportion of NDI patients, significant growth impairment remains (19).

Only 65% of the cohort reported having a pediatric dietitian available for management of such a complicated and rare condition. This may be related to variable access to ancillary health resources in different regions of the U.S. and level of comfort of the primary nephrologist. One focus of this study was the multiple treatment regimens used by pediatric nephrologists in managing children with NDI. It has long been shown that treatment with a combination of thiazide and NSAID initially reduces polyuria and promotes natriuresis (20–24). Due to concerns of gastrointestinal (GI) bleeding secondary to NSAIDs, some practitioners advocate use of a thiazide diuretic with the PSD amiloride. While amiloride has no GI bleeding side effects, it may cause nausea and emesis, which is often transient (22). In our cohort, 33% of children were treated with a thiazide and PSD compared with 15% on a thiazide and NSAID. GI complications and AKI, known adverse effects, were the main reasons for stopping NSAIDS. A significant percentage of our patients had adverse outcomes from their NDI, including CKD stage 2 or higher; hydronephrosis; and/or treatment complications, suggesting the need for prospective trials of various treatment strategies to establish best practices in this population.

While the available evidence seems to favor a combination of thiazide diuretics with PSD when combination therapy is required, there are multiple treatment combinations reported in this cohort (20–24). To date, no comprehensive treatment algorithm has been accepted by the greater pediatric nephrology community in the U.S. Based on data derived from our cohort and results of studies over the past 50 years, we propose the following interventions that merit further evaluation and study. (1) Medical therapy at initial presentation to include thiazides in combination with amiloride should be implemented and studied systematically. Data suggest this yields the most effective lowering of urine volume and preservation of serum potassium with minimal side effects (20, 21). As the child grows much older and toilet training is desired, an NSAID, added to aid in controlling polyuria for successful bladder training, should be studied. (2) Nutritional support at the time of presentation to include management by a renal dietician should be evaluated and specific exploration of low sodium/low solute diet and g-tube placement especially when FTT is present. (3) Urologic studies to include yearly renal ultrasound and post void residuals should be evaluated. An elevated post void residual coupled with abnormal bladder findings merits a referral to a pediatric urologist. (4) Regular renal function monitoring with evaluation of creatinine clearance (CrCl) and vigilance for episodes of AKI should be evaluated, especially as applied during times of inpatient admission or high-risk periods such as dehydration episodes.

Our study had several limitations. The data were collected retrospectively and we were not able to obtain complete data on all of the patients. In addition, we collected data points based on interventions (diagnosis, g-tube placement, last follow-up) and did not collect specified longitudinal data. Furthermore, we did not require a specific length of follow-up in order to contribute data to the study, resulting in varied follow-up time for each patient.

In summary, in our large North American cohort of 66 children with NDI, most children who undergo genetic testing have a defined mutation (70%). Thiazides (74%) and PSD (67%) were employed in the majority of children and combination treatment with multiple agents was common. A significant proportion of children have persistent short stature (38%) and are underweight (29%) at last follow-up and this portends concerns for long-term psycho-social functioning. Adverse events were all too common for these children: hospitalizations (61%), urologic complications (37%), and CKD stage 2 or higher (27%) supporting the need for careful treatment trials to improve outcomes and decrease disease burden in these children.

## Data Availability Statement

Data can be requested by contacting the corresponding author.

## Ethics Statement

The studies involving human participants were reviewed and approved by Connecticut Childrens IRB. Written informed consent from the participants' legal guardian/next of kin was not required to participate in this study in accordance with the national legislation and the institutional requirements.

## Author Contributions

All authors listed have substantial contributions to the conception or design of the work, or the acquisition, analysis, or interpretation of data for the work. Each author listed participated in wither drafting the work or revising it critically for important intellectual content. They have all provided approval for publication of the content.

### Conflict of Interest

The authors declare that the research was conducted in the absence of any commercial or financial relationships that could be construed as a potential conflict of interest.
